# Supplementary material for: De-Speckling Breast Cancer Ultrasound Images Using a Rotationally Invariant Block Matching Based Non-Local Means (RIBM-NLM) Method
Source: Diagnostics (Basel). 2022 Mar 30;12(4):862. doi: 10.3390/diagnostics12040862 (PMC9030862; doi:10.3390/diagnostics12040862)
Supplement: Supplementary file 1 [file diagnostics-12-00862-s001.zip › diagnostics-1620637-supplementary.pdf]

Supplementary Information

SI1. Supplementary Tables

Table S1: Average PSNR, MSE, RMSE, and t(s) for several algorithms using public database images.

**Method**	**σ**											
	**$\sigma = 10$**				**$\sigma = 20$**				**$\sigma = 50$**			
	**PSNR**	**MSE**	**RMSE**	**t(s)**	**PSNR**	**MSE**	**RMSE**	**t(s)**	**PSNR**	**MSE**	**RMSE**	**t(s)**
**NLMF**	65.2498	0.00595	0.07713	175.49344	58.5145	0.07154	0.26748	178.50518	48.0051	0.48261	0.69469	182.43975
**ONLMF**	68.2873	0.005503	0.07418	137.33325	59.5059	0.04150	0.20372	138.93431	49.7482	0.40436	0.63589	140.57655
**SBF**	69.2665	0.005049	0.07106	131.43482	61.7567	0.02483	0.15732	130.96582	51.0262	0.34989	0.59143	132.56860
**Proposed**	72.0042	0.004600	0.06782	80.441520	65.9770	0.01431	0.11911	82.53525	54.0056	0.23502	0.48478	82.56582

Table S2: Averaged quantitative de-noising results for public database images. DI1, public database image1.

**Image**	**σ**					
	**$\sigma = 10$**		**$\sigma = 20$**		**$\sigma = 50$**	
	**PSNR**	**MSE**	**PSNR**	**MSE**	**PSNR**	**MSE**
**DI1**	72.3757	0.004694	66.8663	0.12475	53.479	0.238424
**DI2**	71.9765	0.004616	66.1763	0.125073	54.5441	0.239503

**DI3**	71.8738	0.004697	65.2115	0.129164	53.5035	0.236537
**DI4**	71.5449	0.004613	66.6674	0.12307	54.2076	0.232618
**DI5**	71.6832	0.004619	65.9757	0.120714	53.5946	0.234196
**DI6**	72.1022	0.004568	66.274	0.127537	54.6011	0.234476
**DI7**	71.693	0.004568	65.8826	0.12369	53.3371	0.239749
**DI8**	72.3616	0.004506	66.3864	0.12089	54.3981	0.235122
**DI9**	72.0064	0.004605	65.0454	0.125521	53.8598	0.230858
**DI10**	71.6332	0.004527	66.7051	0.126692	53.886	0.232648
**DI11**	71.537	0.004674	66.2956	0.121662	53.8429	0.232553
**DI12**	72.437	0.004593	66.4715	0.120154	53.3437	0.230727
**DI13**	71.9106	0.00454	65.8342	0.129728	54.3823	0.231607
**DI14**	72.1516	0.004622	65.5268	0.123624	54.2415	0.235119
**DI15**	71.6775	0.004645	65.6163	0.128334	54.3043	0.231523
**DI16**	72.1257	0.004518	65.5379	0.121041	53.7258	0.239916
**DI17**	71.7834	0.00469	65.0826	0.127105	53.191	0.236675
**DI18**	72.4216	0.004643	65.2948	0.124253	53.419	0.233256
**DI19**	72.2519	0.004671	66.8781	0.127759	53.7116	0.231456
**DI20**	72.1712	0.004589	66.4715	0.129709	54.5456	0.239029
**DI21**	71.6727	0.004503	65.6286	0.120461	54.7922	0.239613
**DI22**	72.0086	0.004517	66.13	0.122052	53.1524	0.234233
**DI23**	71.5342	0.004634	66.8032	0.125602	54.1466	0.239477
**DI24**	71.5189	0.00453	66.6035	0.127793	53.5477	0.233938
**DI25**	71.543	0.004553	65.5084	0.123126	54.6155	0.231854
**DI26**	71.8197	0.004648	66.1178	0.121084	54.4207	0.234364
**DI27**	71.8959	0.004629	65.8021	0.126811	53.8762	0.234021
**DI28**	72.3409	0.004623	66.5474	0.121193	54.7208	0.238145
**DI29**	72.0239	0.004544	66.5313	0.123096	54.1443	0.23977
**DI30**	72.3175	0.004654	65.3973	0.12921	53.2391	0.231589
**DI31**	71.6771	0.004633	66.3749	0.124021	54.0243	0.237626
**DI32**	71.6791	0.004665	66.5979	0.12683	54.3528	0.233173

**DI33**	71.5847	0.004641	65.4049	0.124371	54.4969	0.239812
**DI34**	72.3048	0.004638	65.3398	0.127168	54.5287	0.230336
**DI35**	72.3483	0.00456	65.9688	0.129533	53.2643	0.230906
**DI36**	71.65	0.004509	65.0886	0.126302	54.1148	0.234489
**DI37**	72.1126	0.004523	66.1094	0.120766	53.1614	0.235773
**DI38**	71.5262	0.00456	65.7119	0.129196	54.2845	0.239639
**DI39**	71.6764	0.004535	66.9223	0.127745	54.8678	0.233194
**DI40**	72.1513	0.00458	65.2578	0.128247	53.4654	0.234705
**DI41**	72.3118	0.004645	66.2419	0.126586	53.1411	0.238988
**DI42**	72.2458	0.004562	65.3163	0.120227	53.1009	0.239832
**DI43**	72.4054	0.00467	66.3885	0.128331	53.4089	0.235897
**DI44**	72.4351	0.00461	66.7532	0.124097	53.271	0.23104
**DI45**	72.0319	0.004629	65.8127	0.127942	54.7247	0.239648
**DI46**	71.9723	0.004672	66.9412	0.123346	54.5674	0.233541
**DI47**	71.8413	0.004684	65.7204	0.121776	54.9722	0.233112
**DI48**	71.6705	0.0046	66.9272	0.124157	54.6965	0.237478
**DI49**	71.7384	0.004538	65.1444	0.12203	53.6558	0.23848
**DI50**	72.4699	0.004584	66.9737	0.129692	54.3168	0.231433
**DI51**	72.4224	0.004668	66.3263	0.122628	54.5732	0.230789
**DI52**	71.6344	0.004643	65.5081	0.122787	53.7128	0.237061
**DI53**	71.7306	0.004691	65.6619	0.127394	54.4566	0.232108
**DI54**	72.4662	0.004671	66.9625	0.122269	53.9776	0.2347
**DI55**	72.1817	0.004632	65.053	0.123544	53.4902	0.237389
**DI56**	72.2638	0.004606	66.4157	0.12114	54.018	0.231551
**DI57**	71.8213	0.004699	65.3127	0.125612	54.4904	0.232632
**DI58**	72.4414	0.004539	65.533	0.128587	53.1645	0.232572
**DI59**	71.6964	0.004576	66.44	0.12883	53.5936	0.233541
**DI60**	72.3876	0.004575	65.8803	0.122168	53.9059	0.231517
**DI61**	72.0016	0.004586	66.2989	0.12515	54.6082	0.230285
**DI62**	72.2617	0.00464	65.5367	0.129356	53.1776	0.234006

**DI63**	72.1572	0.004603	66.9873	0.12051	54.2475	0.238848
**DI64**	72.2107	0.004533	66.4522	0.120353	53.3444	0.237538
**DI65**	72.4764	0.004639	65.5176	0.122684	54.7435	0.230927
**DI66**	71.6194	0.004697	65.4782	0.123169	54.7441	0.239224
**DI67**	71.6767	0.004533	66.6827	0.123578	54.5959	0.239521
**DI68**	71.8044	0.004601	65.0187	0.124237	54.1729	0.237838
**DI69**	72.447	0.004548	65.7099	0.127534	53.6226	0.233357
**DI70**	71.9914	0.00461	65.8575	0.124933	54.5356	0.230849
**DI71**	71.8755	0.004509	66.8214	0.122713	54.314	0.237427
**DI72**	72.3331	0.004667	66.6213	0.126348	53.9064	0.230358
**DI73**	71.7158	0.00453	65.2198	0.122899	53.8748	0.233946
**DI74**	71.8892	0.004516	66.3979	0.12874	53.0888	0.238821
**DI75**	71.5232	0.00457	65.6426	0.120942	54.6868	0.233928
**DI76**	71.6502	0.004655	65.6194	0.125954	54.5868	0.237014
**DI77**	72.4321	0.004507	66.4071	0.127102	53.5365	0.232022
**DI78**	72.4394	0.004631	66.291	0.121737	54.8755	0.234947
**DI79**	72.1346	0.004677	66.3936	0.121215	53.9766	0.232425
**DI80**	71.9122	0.004567	65.464	0.122575	54.3867	0.237536
**DI81**	72.3143	0.004582	65.7886	0.123204	54.9681	0.231465
**DI82**	72.0595	0.00463	66.2714	0.129067	53.4552	0.234839
**DI83**	72.4757	0.004636	65.9426	0.127784	54.5088	0.234607
**DI84**	72.1717	0.004504	66.6765	0.123456	53.5301	0.233338
**DI85**	72.0429	0.004582	65.244	0.127046	54.8177	0.238375
**DI86**	71.931	0.004517	66.4957	0.121571	53.6235	0.233378
**DI87**	72.205	0.004696	66.4513	0.126924	54.3201	0.237554
**DI88**	71.9345	0.004566	66.0521	0.12272	54.9688	0.234712
**DI89**	72.3163	0.004519	65.4463	0.122525	53.7127	0.23824
**DI90**	72.4467	0.004595	65.281	0.127827	53.6775	0.236475
**DI91**	72.252	0.00451	65.6316	0.125601	54.054	0.231076
**DI92**	72.4712	0.0047	66.5946	0.127293	53.1168	0.237468

**DI93**	72.4502	0.004593	66.7787	0.127448	54.5104	0.233744
**DI94**	72.4478	0.004582	65.7328	0.121197	53.2188	0.235817
**DI95**	72.0672	0.004639	66.3333	0.127429	54.843	0.237448
**DI96**	72.4204	0.00462	65.6266	0.123238	54.0274	0.237933
**DI97**	71.7291	0.004657	65.0108	0.121637	54.7675	0.239208
**DI98**	72.0765	0.004522	65.4004	0.122362	54.0734	0.238928
**DI99**	71.706	0.004551	66.1047	0.125809	53.542	0.238603
**DI100**	72.2887	0.004652	66.1617	0.123329	53.6484	0.237341
**DI101**	72.4853	0.004669	66.286	0.123398	54.4446	0.23229
**DI102**	72.0889	0.004613	65.1459	0.126438	54.7799	0.236992
**DI103**	72.0535	0.004543	65.4592	0.125839	54.9658	0.233482
**DI104**	71.5109	0.004522	65.5042	0.122664	54.2053	0.238687
**DI105**	71.6151	0.004647	66.4206	0.129636	53.3592	0.235972
**DI106**	71.9458	0.004565	65.5434	0.125241	53.4897	0.236433
**DI107**	72.0474	0.004578	66.7666	0.129948	54.194	0.234166
**DI108**	72.1101	0.004671	66.2201	0.122958	54.9683	0.233557
**DI109**	72.4231	0.004621	66.253	0.120748	54.9916	0.23999
**DI110**	72.3272	0.004668	65.5124	0.126266	53.1329	0.236625
**DI111**	71.6013	0.004656	65.0665	0.124426	53.1432	0.231221
**DI112**	72.4325	0.004599	66.5461	0.122272	54.288	0.23637
**DI113**	71.7981	0.004536	65.5794	0.123509	53.0626	0.234387
**DI114**	71.6509	0.004553	65.0829	0.123296	53.2283	0.23427
**DI115**	71.8947	0.004518	65.075	0.125063	53.0293	0.237819
**DI116**	71.6769	0.004508	65.187	0.127297	53.9101	0.239012
**DI117**	71.7892	0.004595	65.5839	0.12731	53.5495	0.233896
**DI118**	71.7726	0.004654	66.4422	0.128122	53.8317	0.23967
**DI119**	71.7091	0.004516	66.3272	0.121313	53.7219	0.23617
**DI120**	72.1454	0.00458	66.9744	0.12052	54.2918	0.238032
**DI121**	72.4215	0.004629	66.8069	0.124033	54.9885	0.233323
**DI122**	72.3134	0.004553	66.351	0.128403	54.4543	0.236377

**DI123**	72.3933	0.00458	65.1425	0.120972	53.5311	0.23196
**DI124**	71.7874	0.004564	65.7153	0.121973	53.8814	0.237168
**DI125**	71.751	0.004648	65.1307	0.129205	53.463	0.232484
**DI126**	71.5972	0.004666	65.0937	0.12398	53.1464	0.232796
**DI127**	71.6586	0.004684	65.4948	0.129325	53.1832	0.235236
**DI128**	71.9118	0.004649	65.2877	0.128996	54.6263	0.234074
**DI129**	72.1844	0.004655	66.3159	0.126384	54.8737	0.235798
**DI130**	72.317	0.004588	65.6062	0.121705	53.5109	0.234748
**DI131**	72.066	0.00452	66.1261	0.1219	54.8312	0.238468
**DI132**	71.56	0.004649	66.6671	0.12451	54.4516	0.237431
**DI133**	71.8345	0.0047	66.1485	0.125692	53.375	0.233086
**DI134**	71.8699	0.004501	65.052	0.121306	54.9531	0.239684
**DI135**	72.1159	0.004536	65.0044	0.123538	54.1231	0.230273
**DI136**	71.7003	0.004685	65.1589	0.121605	53.6921	0.234857
**DI137**	72.4772	0.004642	66.5459	0.126326	53.0028	0.233978
**DI138**	72.2031	0.004677	66.2853	0.128194	54.2195	0.231771
**DI139**	72.1155	0.004674	65.5358	0.125758	53.285	0.239487
**DI140**	72.4489	0.004559	65.8759	0.121962	53.47	0.2342
**DI141**	71.7686	0.004514	65.1584	0.124896	54.2799	0.232722
**DI142**	72.4702	0.004524	65.9989	0.126127	54.6199	0.237867
**DI143**	71.5459	0.004682	66.0168	0.125931	53.3809	0.239607
**DI144**	72.2566	0.004547	66.4836	0.126336	54.9129	0.237933
**DI145**	71.8115	0.004524	65.8242	0.122895	54.2026	0.233963
**DI146**	71.8624	0.004662	66.281	0.120288	54.8009	0.23089
**DI147**	71.5852	0.004695	66.8775	0.125241	53.5937	0.2359
**DI148**	71.5	0.004561	66.3992	0.126947	53.2717	0.237154
**DI149**	72.1646	0.004622	66.0192	0.120987	53.5081	0.235418
**DI150**	71.5895	0.004611	65.6556	0.12981	54.5632	0.235941
**DI151**	71.6386	0.004691	65.0238	0.122548	54.7161	0.232695
**DI152**	71.6661	0.004559	66.7847	0.122063	54.4428	0.237238

**DI153**	71.943	0.004519	66.8653	0.120412	54.5737	0.23461
**DI154**	71.7268	0.004677	65.9834	0.125744	54.3036	0.236581
**DI155**	72.2013	0.004677	66.1019	0.120485	53.6103	0.239692
**DI156**	72.1304	0.004672	66.0157	0.123224	53.5335	0.239913
**DI157**	72.3459	0.004689	66.9232	0.120475	53.4701	0.234894
**DI158**	72.3556	0.004699	65.0719	0.121261	53.6376	0.235504
**DI159**	71.834	0.004547	66.7958	0.122311	54.0869	0.232202
**DI160**	72.2332	0.004628	66.7277	0.123867	54.7832	0.237916
**DI161**	72.3284	0.004684	66.2549	0.129035	54.8897	0.232159
**DI162**	72.2766	0.004591	66.0239	0.120151	53.1027	0.231307
**DI163**	71.9145	0.004571	65.6341	0.127241	54.6769	0.230407
**DI164**	71.8192	0.004598	65.8155	0.120753	53.8338	0.237913
**DI165**	71.5525	0.00454	65.6407	0.127443	54.7664	0.231608
**DI166**	72.1874	0.004545	66.8172	0.128599	54.9381	0.237722
**DI167**	71.9118	0.004692	65.8401	0.126661	53.933	0.230176
**DI168**	71.6915	0.004588	66.9958	0.12565	53.2418	0.230581
**DI169**	71.5638	0.004557	66.2687	0.127046	53.0856	0.235816
**DI170**	72.1939	0.004531	65.9435	0.123322	54.4145	0.235012
**DI171**	72.0493	0.004658	66.7644	0.121539	53.8504	0.237362
**DI172**	71.6745	0.004527	66.3986	0.126959	53.3632	0.237756
**DI173**	72.3292	0.004567	66.454	0.125013	53.5794	0.230604
**DI174**	71.6934	0.004578	65.3863	0.12956	53.1766	0.235495
**DI175**	71.6141	0.004622	65.4482	0.125253	53.1471	0.23864
**DI176**	71.5382	0.004597	66.0349	0.128119	54.5958	0.232304
**DI177**	71.9714	0.004693	65.2956	0.12928	54.5628	0.232922
**DI178**	71.774	0.004522	66.0903	0.125623	53.2857	0.236306
**DI179**	71.9381	0.004617	65.207	0.128101	53.8561	0.234523
**DI180**	71.6988	0.00469	65.3986	0.129823	53.1981	0.232358
**DI181**	71.8168	0.004562	65.5001	0.12743	53.705	0.23405
**DI182**	71.7458	0.004559	65.0523	0.121743	53.2566	0.234914

**DI183**	71.9822	0.00456	65.211	0.127492	54.1055	0.239727
**DI184**	72.3455	0.004648	65.1563	0.12979	54.714	0.236332
**DI185**	72.2676	0.004609	66.612	0.129674	53.337	0.232422
**DI186**	72.3403	0.004552	65.6063	0.123491	53.3734	0.238963
**DI187**	72.0076	0.004547	65.4415	0.123262	54.4914	0.231801
**DI188**	72.2849	0.004612	66.337	0.127501	53.3932	0.237716
**DI189**	71.5745	0.004627	65.3548	0.121594	53.3119	0.235532
**DI190**	71.6637	0.004642	66.3836	0.122209	53.3805	0.234199
**DI191**	71.537	0.004558	66.8547	0.124121	54.4671	0.230762
**DI192**	71.595	0.004553	65.1114	0.129151	53.6427	0.232448
**DI193**	72.3353	0.004622	66.1201	0.120676	54.7564	0.230834
**DI194**	72.2393	0.004612	66.6189	0.127188	54.3429	0.232313
**DI195**	72.4813	0.004585	65.8121	0.12965	54.665	0.237693
**DI196**	72.3953	0.004658	65.2658	0.121811	53.7896	0.235072
**DI197**	72.4586	0.004593	65.0912	0.122124	54.1955	0.23691
**DI198**	72.1487	0.00465	66.7421	0.120913	54.9758	0.237855
**DI199**	72.2604	0.00468	66.0545	0.124899	54.8577	0.235606
**DI200**	72.1627	0.00451	65.2323	0.125598	53.3514	0.236679
**DI201**	72.4988	0.004549	66.1223	0.122685	53.9694	0.234209
**DI202**	71.5032	0.004564	66.4624	0.127644	54.7106	0.236451
**DI203**	72.4951	0.004591	66.1232	0.121222	53.5931	0.237296
**DI204**	72.0172	0.004695	65.8243	0.128801	53.644	0.238296
**DI205**	72.0233	0.00452	66.5634	0.122648	54.4046	0.234542
**DI206**	71.904	0.004597	66.8505	0.128755	53.1436	0.239395
**DI207**	71.7962	0.004609	65.1335	0.129974	54.8363	0.23319
**DI208**	71.971	0.004509	65.7958	0.122641	54.9499	0.231391
**DI209**	72.4303	0.004578	65.8638	0.120831	54.5668	0.238514
**DI210**	71.5212	0.004569	66.0192	0.124567	54.9775	0.230765
**DI211**	72.2541	0.004664	65.775	0.12199	54.8678	0.235843
**DI212**	71.8245	0.004643	66.8543	0.123915	53.5615	0.236306

**DI213**	71.893	0.00459	65.5149	0.121622	54.8039	0.234604
**DI214**	71.6995	0.00451	66.6108	0.12859	53.8572	0.234679
**DI215**	71.7693	0.004672	66.9084	0.123413	54.461	0.238539
**DI216**	71.8667	0.004681	66.7157	0.12684	53.0809	0.233274
**DI217**	71.8412	0.00465	65.3799	0.123249	53.2648	0.230082
**DI218**	72.3387	0.004648	66.8669	0.122192	53.4509	0.2333
**DI219**	72.1793	0.004546	66.9188	0.120254	53.1823	0.232063
**DI220**	72.2295	0.004501	65.2932	0.12932	54.9414	0.238154
**DI221**	71.6146	0.004667	65.7635	0.121667	53.082	0.236978
**DI222**	71.6377	0.00452	65.3392	0.127664	53.8303	0.239511
**DI223**	71.6638	0.00455	66.8927	0.128537	54.2812	0.238607
**DI224**	72.2555	0.004536	65.878	0.123931	54.7371	0.233779
**DI225**	72.2289	0.004658	65.7855	0.128916	53.492	0.231866
**DI226**	71.6763	0.004661	65.2908	0.122564	54.027	0.230447
**DI227**	72.4066	0.004507	66.6295	0.123412	54.4028	0.232256
**DI228**	71.7208	0.00468	65.2585	0.129332	53.8213	0.231469
**DI229**	71.7848	0.004514	65.1785	0.125432	53.0226	0.230812
**DI230**	71.9253	0.00461	66.639	0.125891	53.1809	0.237667
**DI231**	71.51	0.004597	66.1488	0.124921	54.2331	0.237047
**DI232**	72.2186	0.004603	65.9073	0.127594	54.6147	0.233677
**DI233**	71.7654	0.004518	65.6823	0.128015	54.1198	0.231958
**DI234**	71.8532	0.004632	65.4463	0.127274	54.5667	0.230288
**DI235**	71.5659	0.004541	65.6008	0.125796	54.4749	0.23696
**DI236**	72.1919	0.004513	66.7915	0.129992	54.1104	0.23112
**DI237**	72.4814	0.004599	66.6474	0.124158	54.9939	0.236776
**DI238**	72.4393	0.00454	66.4038	0.128339	53.5438	0.237813
**DI239**	71.7175	0.00457	65.1521	0.128857	53.8869	0.238863
**DI240**	72.4515	0.004593	66.5637	0.127348	53.1696	0.233621
**DI241**	72.1811	0.00453	66.7293	0.126187	53.639	0.230915
**DI242**	72.0037	0.004523	66.401	0.120563	53.9941	0.238249

**DI243**	71.7885	0.004695	65.1228	0.12446	54.4201	0.230236
**DI244**	71.5796	0.00451	66.2013	0.125179	53.3587	0.230293
**DI245**	71.5796	0.004561	65.8743	0.120892	53.1232	0.236982
**DI246**	72.3572	0.004648	66.5785	0.123525	54.604	0.23074
**DI247**	72.1511	0.004661	66.5688	0.120901	54.2889	0.232384
**DI248**	71.9826	0.004613	65.5704	0.129021	54.1246	0.232017
**DI249**	72.4765	0.004676	65.0736	0.126175	53.7892	0.236388
**DI250**	72.3575	0.00467	66.1393	0.124331	53.6833	0.230434
**Average**	**72.0042**	**0.004600**	**65.9770**	**0.124915**	**54.0056**	**0.235024**

Table S3: Average PSNR, MSE, RMSE and t(s) for several algorithms using private image dataset.

**Method**	**σ**											
	**$\sigma = 10$**				**$\sigma = 20$**				**$\sigma = 50$**			
	**PSNR**	**MSE**	**RMSE**	**t(s)**	**PSNR**	**MSE**	**RMSE**	**t(s)**	**PSNR**	**MSE**	**RMSE**	**t(s)**
**NLMF**	65.4445	0.00797	0.08928	179.99630	59.1329	0.08648	0.29381	181.89353	48.6903	0.38149	0.60935	183.18329
**ONLMF**	68.0621	0.00589	0.07669	138.30341	61.2797	0.05243	0.22863	139.21338	50.9309	0.52901	0.72660	140.67373
**SBF**	70.1315	0.00415	0.06425	132.02910	63.0977	0.02710	0.16442	133.99915	51.7781	0.45485	0.67402	131.83787
**Proposed**	72.1528	0.00341	0.05835	81.45873	66.2639	0.01559	0.12450	82.82760	54.9143	0.24245	0.29381	83.75906

SI2. Supplementary Figures

a b

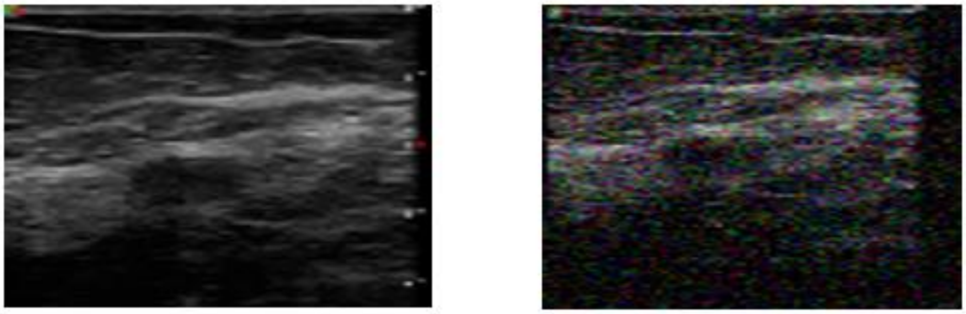

Figure S1: Illustration of blurring with Gaussian noise $\sigma = 20$. (a) Original image. (b) Blurred image.

a b

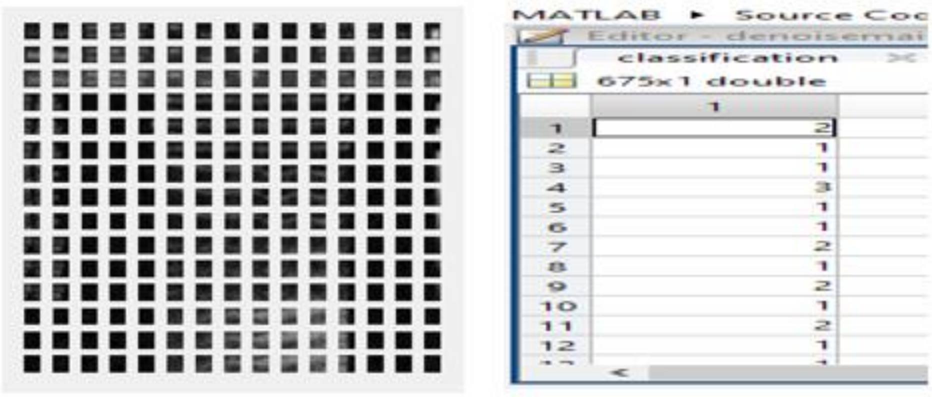

Figure S2**:** Clustering based pre-classification. (a) Patch separation. (b) K-means clustering.

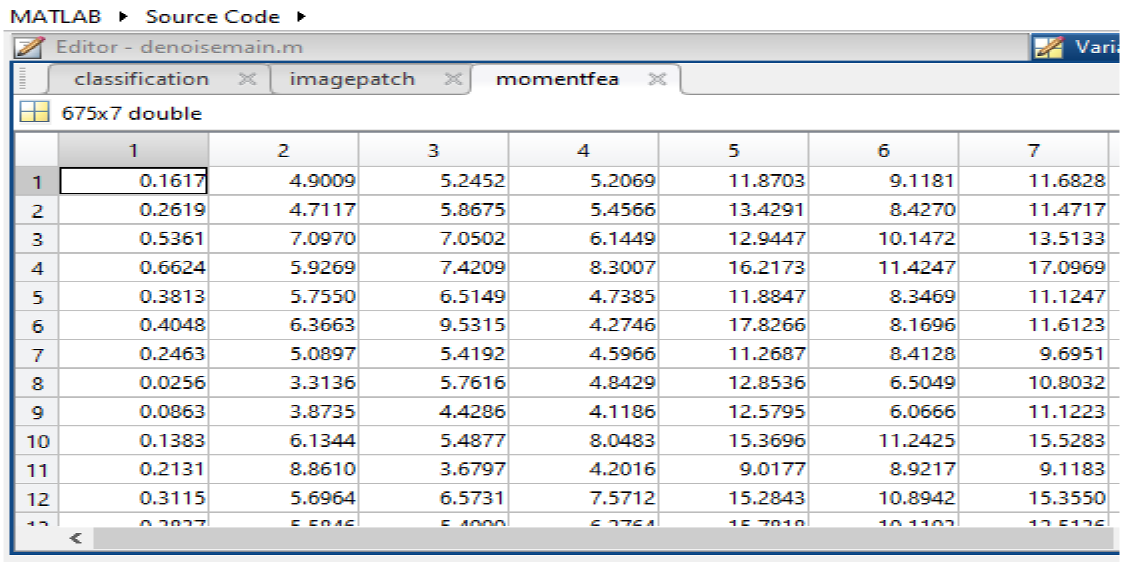

	1	2	3	4	5	6	7
1	0.1617	4.9009	5.2452	5.2069	11.8703	9.1181	11.6828
2	0.2619	4.7117	5.8675	5.4566	13.4291	8.4270	11.4717
3	0.5361	7.0970	7.0502	6.1449	12.9447	10.1472	13.5133
4	0.6624	5.9269	7.4209	8.3007	16.2173	11.4247	17.0969
5	0.3813	5.7550	6.5149	4.7385	11.8847	8.3469	11.1247
6	0.4048	6.3663	9.5315	4.2746	17.8266	8.1696	11.6123
7	0.2463	5.0897	5.4192	4.5966	11.2687	8.4128	9.6951
8	0.0256	3.3136	5.7616	4.8429	12.8536	6.5049	10.8032
9	0.0863	3.8735	4.4286	4.1186	12.5795	6.0666	11.1223
10	0.1383	6.1344	5.4877	8.0483	15.3696	11.2425	15.5283
11	0.2131	8.8610	3.6797	4.2016	9.0177	8.9217	9.1183
12	0.3115	5.6964	6.5731	7.5712	15.2843	10.8942	15.3550
[illegible]	[illegible]	[illegible]	[illegible]	[illegible]	[illegible]	[illegible]	[illegible]

Figure S3: Moment invariants.

	1	2	3	4
1	1.2713	0.0165	0.0502	[illegible]

Figure S4: Illustration of rotation angles calculated.
